# Comparison of combined application treatment with one-visit varnish treatments in an orthodontic population

**DOI:** 10.4317/medoral.18261

**Published:** 2013-02-05

**Authors:** Ozgul Baygin, Tamer Tuzuner, Mehmet B. Ozel, Ozge Bostanoglu

**Affiliations:** 1 PhD. Karadeniz Technical University, Faculty of Dentistry, Department of Pediatric Dentistry; 2 PhD. Karadeniz Technical University, Faculty of Dentistry, Department of Orthodontics; 3 Research Asisstant. Karadeniz Technical University, Faculty of Dentistry, Department of Orthodontics

## Abstract

Objective: To evaluate the effect of chlorhexidine-thymol varnish alone, its combination with chlorhexidine-fluoride containing dentifrice and fluoride varnish on oral hygiene and caries prevention in orthodontic patients. Study design: Sixty patients, aged 12-18, with orthodontic fixed appliances were randomly assigned into three groups as follows: Group 1 (n=20): 1% chlorhexidine and 1% thymol varnish (Cervitec®Plus); Group 2 (n=20): Cervitec®Plus+ 0.2% chlorhexidine and 0.2% sodium fluoride (900 ppm fluoride) (Cervitec®Gel)); and Group 3 (n=20): 0.1% fluoride varnish (Fluor Protector®). Mutans streptococci (MS), lactobacilli (LB) levels, buffering capacity (BC), visible plaque index (VPI), and gingival bleeding index (GBI) scores were evaluated at four stages: T0, before orthodontic bonding; T1, one week after orthodontic bonding; T2, one week; and T3, four weeks after the first application, respectively. Inter and intra group comparisons were made by the Kruskal-Wallis, Mann-Whitney U, Friedman and Wilcoxon Signed-Rank tests with Bonferroni step-down correction (P<0.017). Results: Significantly lower MS and LB levels were found in Group 2 than Group 1 (T2) and 3 (T2, T3) (P<0.017). Groups 1-2 (T2) showed significantly higher BC (P<0.017) and lower VPI and GBI (P<0.017) scores compared with Group 3. Decreased MS levels at T2 (P<0.017) and T3 (P>0.017) were found in Group1-2 compared with T0. Significantly lower LB levels were recorded in Group 2 at T2 compared with T0 (P<0.017) while no significant differences were seen in Group 1 and 3 (P>0.017). Conclusions: Addition of Cervitec®Plus+Cervitec®Gel combination to the standard oral hygiene regimen may be beneficial for orthodontic patients for maintaining oral health by reducing bacterial colonisation and gingivitis.

** Key words:**Chlorhexidine, flouride, mutans streptococci, lactobacilli, antibacterial effect, plaque, gingivitis, orthodontic treatment.

## Introduction

Providing proper oral hygiene during fixed orthodontic therapy is challenging. Fixed orthodontic appliances modify the oral flora and reduce the potential benefits of brushing and salivary flow on plaque (1,2). The increased amount of plaque adjacent to bands and brackets commonly results in poor oral hygiene and changes in the oral flora, including increased levels of *Mutans streptococci* (MS) and *Lactobacilli* (LB), increased plaque and bleeding scores, and reduced levels of buffe-ring capacity (3-5). Insufficient removal of supragingival plaque may cause development of white spot lesions and gingival diseases (3,6).

Preventive programs should be implemented to avoid the potentially detrimental consequences of fixed orthodontic appliances in the oral environment (1). The application of antimicrobial therapies with common commercially produced antibacterial agents, such as varnishes, mouthwashes, or dentifrice gel formulations, particularly with chlorhexidine (CHX), have been found to be a beneficial preventive strategy for reducing undesirable outcomes of fixed orthodontic appliances such as peridontal problems and white spot lesions (1,5,7). Several studies investigated the effects of various concentrations of chlorhexidine on oral microflora (1,4,8). It was shown that with a concentration as low as 0.2%, chlorhexidine is an effective antimicrobial agent to reduce *Streptococcus Mutans* levels in oral flora (4).

Chlorhexidine and thymol (Cervitec®Plus) combination as a varnish treatment was found to be beneficial in inhibiting salivary MS levels and reducing gingivitis therefore improves oral hygiene in orthodontic patients (8,9-11). Chlorhexidine-based toothpastes as an adjunct to mechanical cleaning facilitate the removal of plaque and providing better oral hygiene in orthodontic patients (1,10).

Topical fluoride application by toothpastes, gels, rinses, and varnishes were found to be beneficial in patients with fixed appliances (6). However, a fluoride varnish regimen alone was postulated as an ineffective effort to improve oral hygiene status of orthodontic patients (11).

Therefore, fluoride varnishes were suggested to be used in combination with other forms of fluoride supplements (12). Additionally, a combined antimicrobial strategy with antibacterial agents and fluoride has been considered a more effective way to reduce possible caries-associated problems in orthodontic patients who undergo fixed appliance therapy (5). Moreover, a recently developed gel formulation was introduced with a combination of 0.2% chlorhexidine and 0.2% sodium fluoride (Cervitec®Gel).

The main aim of this study was to investigate the antibacterial effectiveness of chlorhexidine-thymol varnish (Cervitec®Plus) alone, its combination with chlorhexidine-fluoride containing dentifrice (Cervitec®Gel) and fluoride varnish (Fluor Protector®) by measuring salivary *Mutans streptococci* and *Lactobacilli* levels and buffering capacity, visible plaque index and gingival bleeding index scores in patients who underwent orthodontic fixed appliance therapy.

## Material and Methods

This research was designed as a prospective randomized and blind clinical study. Individuals who were scheduled to start their fixed orthodontic treatment at the orthodontic clinic of Faculty of Dentistry, Karadeniz Technical University, were included. The patients or their parents were informed about the study and signed informed consent forms. Ethical approval was taken from The Karadeniz Technical University Ethics Committee before the study was initiated (2011/36).

The selection criteria for inclusion in the study were as follows: Patients with complete permanent dentition, good general health and no use of pharmacotherapics for the last three months and aged between 12 to 18 at the initiation of fixed appliance therapy were included in the study. All the individuals underwent fully banded, non-extraction edgewise treatment. There was no evidence of decalcification on the teeth. There was no known hypersensitivity to chlorhexidine and fluoride. No restorations on the anterior teeth were present. All volunteers presented a minimum level of preexisting gingivitis and plaque.

Sixty patients were included in the study. Patients were advised to use fluoride-free dentifrice, toothbrushing and flossing for two weeks at home before bonding of the brackets. The bonding procedure was as follows: All buccal surfaces of the teeth to be bonded were pumiced with a rubber polishing cup using a low speed handpiece. Then the teeth were rinsed with water, air dried and etched with 37 % orthophosphoric acid for 30 seconds. The acid was rinsed off and the teeth were dried until the enamel exhibited an ice frosted appearance. Transbond XT primer (3M Unitek) was applied on the etched enamel surface and air thinned. Finally .022 edgewise brackets were placed on the teeth with an appropriate amount of Transbond XT (3M Unitek) applied on the bracket base. Excess adhesive around the brackets was removed with a scaler and light cured for 15 seconds with LED (Elipar Freelight 2, 3M Espe, Seefeld, Germany). After bracket bonding, comprehensive oral hygiene instructions were given to the patients by the treating orthodontist (initials of the treating Ortho here probably OB).

Patients were randomly allocated into three groups

-Group 1 (n=20): 1% chlorhexidine diacetate and 1% thymol (Cervitec®Plus, Ivoclar Vivadent, Schaan, Liechtenstein). The individuals received a Cervitec®Plus varnish application one week after bonding of the brackets.

-Cervitec®Plus application procedures were as follows: the tooth surfaces were cleaned thoroughly dried with an air syringe and isolated with cotton rolls. Three drops of Cervitec®Plus were poured into a dappen dish. A thin coat of varnish was applied by means of a Vivadent applicator on all of the surfaces of the bonded teeth (buccal, lingual, occlusal, and proximal areas). The varnish was dispersed with air and allowed to dry and the cotton rolls were removed after 30 seconds. Patients were instructed not to rinse, eat/drink or brush for one hour. They were also told to use fluoride-free dentifrices for four weeks at home. Parents were also told not to provide any other oral hygiene products. A paediatric dentist called the parents and children (every second day) to motivate them in order to provide valid participation in the trial.

-Group 2 (n=20): 1% chlorhexidine diacetate and 1% thymol (Cervitec®Plus Ivoclar Vivadent, Schaan, Liechtenstein) plus 0.2% chlorhexidine digluconate and 0.2% sodium fluoride (900 ppm fluoride), (Cervitec®Gel Ivoclar Vivadent, Schaan, Liechtenstein). The individuals received a Cervitec®Plus varnish application one week after bonding of the brackets. The application procedure of the Cervitec®Plus was the same as Group 1. After the application of the varnish, patients were told not to eat/drink or brush for one hour. In this group, the patients were instructed to brush their teeth with Cervitec®Gel as a dentifrice for four weeks at home. They were instructed to brush their teeth three times a day for 2 min. Parents were also told not to provide any other fluoride-containing dentifrices or oral hygiene products.

-Group 3 (n=20): 5 wt% difluorosilane corresponding to 0.7 wt% F-, (Fluor Protector®, Ivoclar Vivadent, Schaan, Liechtenstein). The individuals received a Fluor Protector® varnish application one week after bracket bonding.

-Fluor Protector® application procedure was as follows: The tooth surface was cleaned thoroughly and dried with an air syringe and isolated with cotton rolls. A thin layer of Fluor Protector® was applied on all tooth surfaces (buccal, lingual, occlusal, and proximal areas) using a suitable single-use applicator (Vivabrush G). The varnish was dispersed with air and allowed to dry, and cotton rolls were removed after 60 seconds. Patients were instructed not to rinse their mouth. After the application of varnish, patients were told not to eat/drink or brush for 45 minutes. The patients were told to use fluoride-free dentifrices for four weeks at home. Parents were also told not to provide any other oral hygiene products. In order to assess plaque accumulation the following parameters were analysed: Salivary *Mutans streptococci* (MS) and *Lactobacilli* (LB) levels and buffering capacity (BC), visible plaque index (VPI), and gingival bleeding index (GBI).

-Bacteria in saliva: CRT® bacteria (CRT® Intro Pack - Caries Risk Test, Ivoclar Vivadent, Schaan, Liechtenstein) was used to determine the MS and LB count in saliva by means of selective culture media.

**Figure 1:**
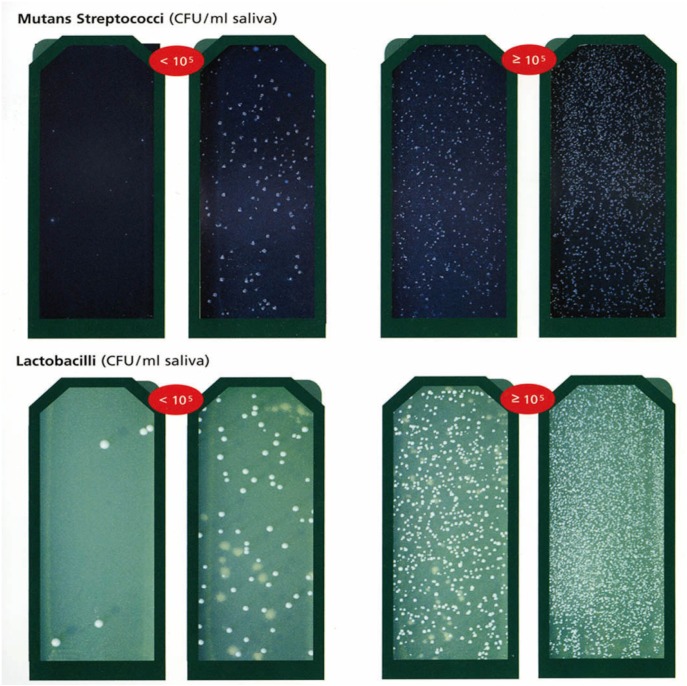
CRT® bacteria (CRT® Intro Pack - Caries Risk Test, Ivoclar Vivadent, Schaan, Liechtenstein): Bright agar surface: determination of LB count in saliva, Blue agar surface: determination of MS count in saliva. The columns in the left indicated less than 105 CFU shows low caries risk and the columns in the right indicated more than 105 CFU shows higher caries risk both for MS and LB per ml in saliva.

-CRT® bacteria procedure: Salivary secretion was stimulated in the patients by chewing an enclosed paraffin pellet and saliva specimens were collected in a suitable container. The agar was removed from the test vial. A NaHCO3 tablet was placed at the bottom of the vial. The protective foils were carefully removed from the two agar surfaces. Both agar surfaces were wetted with saliva using a pipette without scratching the agar surface. The test vial was placed upright in the incubator (Cultura/ Ivoclar Vivadent Schaan, Liechtenstein) and incubated at 37 °C/ 99 °F for 48 hours. After removal of the vial from the incubator, the density of the MS and LB colonies were compared with the corresponding evaluation pictures in the enclosed model chart. Findings of 105 CFU (colony-forming units) or more of MS and LB per ml saliva indicated a high caries risk (Fig. 1).

**Figure 2:**
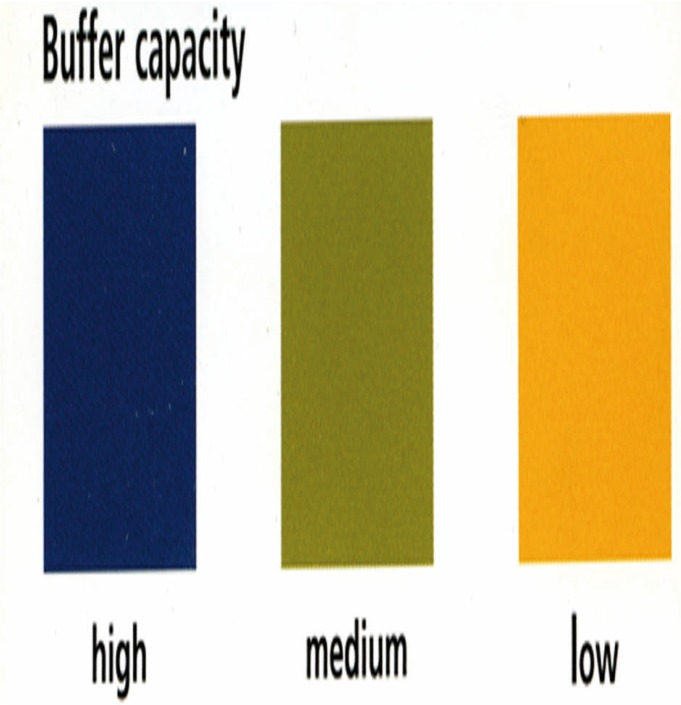
CRT® buffer (CRT® Intro Pack - Caries Risk Test, Ivoclar Vivadent, Schaan, Liechtenstein): Blue indicated a high, green indica-ted a medium, and yellow indicated a low buffer capacity of saliva.

-CRT® buffer procedure: Patients were instructed not to eat or drink anything, chew chewing gum, smoke, brush their teeth, or use any mouthwash for at least one hour before the test was conducted. The patients sat upright in a relaxed position. Salivation was stimulated by having the patient chew a paraffin pellet. The saliva was collected in a calibrated container. The entire yellow test field was wetted with saliva using a pipette. To determine the buffer capacity of saliva, the colour of the test field was compared with the colour samples after exactly five minutes of reaction time (Fig. 2).

-Plaque: The visible plaque index (VPI) was recorded as “1” for visible plaque and “0” for nonvisible plaque on the mesiobuccal surface of every bonded tooth after rinsing and drying the tooth surface. The number of surfaces with plaque was divided by the total number of examined surfaces.

-Gingivitis: The Gingival Bleeding Index (GBI) was assessed by using a 0.5 mm diameter periodontal probe. The gingiva was lightly air-dried, and the probe was gently inserted into the gingival crevice parallel to the long axis of the tooth until slight pressure was felt. At this point, the probe was run along the crevice in contact with the sulcular epithelium. Minimum axial force was used to avoid undue penetration into the tissue, and the probe was moved around the crevice, gently stretching the epithelium. Only the gingival margin at the mesiolabial surfaces was evaluated for every bonded tooth. Bleeding was recorded as “1” and no bleeding as “0”. The number of elicited bleeding points was added up and divided by the number of sites probed.

All of the above-mentioned recordings and measurements were collected and/or measured by two experienced operators and assessor blinding was ensured throughout the trial period. One of the examiners collected the saliva samples for bacteria and executed the buffering capacity procedures. This examiner performed all of the procedures according to the instructions for CRT® bacteria and CRT® buffer procedures for an individual patient. He also recorded all of the measurements for each stage of the study for each individual patient (T0, T1, T2, and T3). The other examiner measured the VPI and GBI values (T0, T1, T2, and T3).

Staining of the teeth and temporary bitter taste problems were also recorded.

These parameters were evaluated at four different time points:

T0 (Baseline): The first samples and measurements were taken just before the bonding procedure. T1 samples and measurements were taken one week after bracket bonding prior to varnish application. Also the varnishes were applied at this stage. T2 samples and measurements were obtained one week after application of varnish.T3 samples and measurements were taken four weeks after application of varnish.

-Statistical analysis

Statistical analyses were performed with the statistical package SPSS 14.0 for Windows (SPSS Inc., Chicago, IL, USA).

Age differences between groups were analyzed with one-way ANOVA and the gender differences were subjected to the Chi-square tests at the significance level of P<0.05.

Kruskal-Wallis and Mann-Whitney U tests with Bonferroni step down correction for inter group comparisons was used. Intra group comparisons between the T0 and other periods (T0- T1, T0- T2 and T0- T3), were assessed by the Friedman and Wilcoxon Signed-Rank test conducted with a Bonferroni correction. The significance level was set as P<0.017.

## Results

A total of 60 children were evaluated in this study. No significant differences were found among three groups regarding demographic properties (age, gender; P>0.05,  Table 1).

**Table 1:**
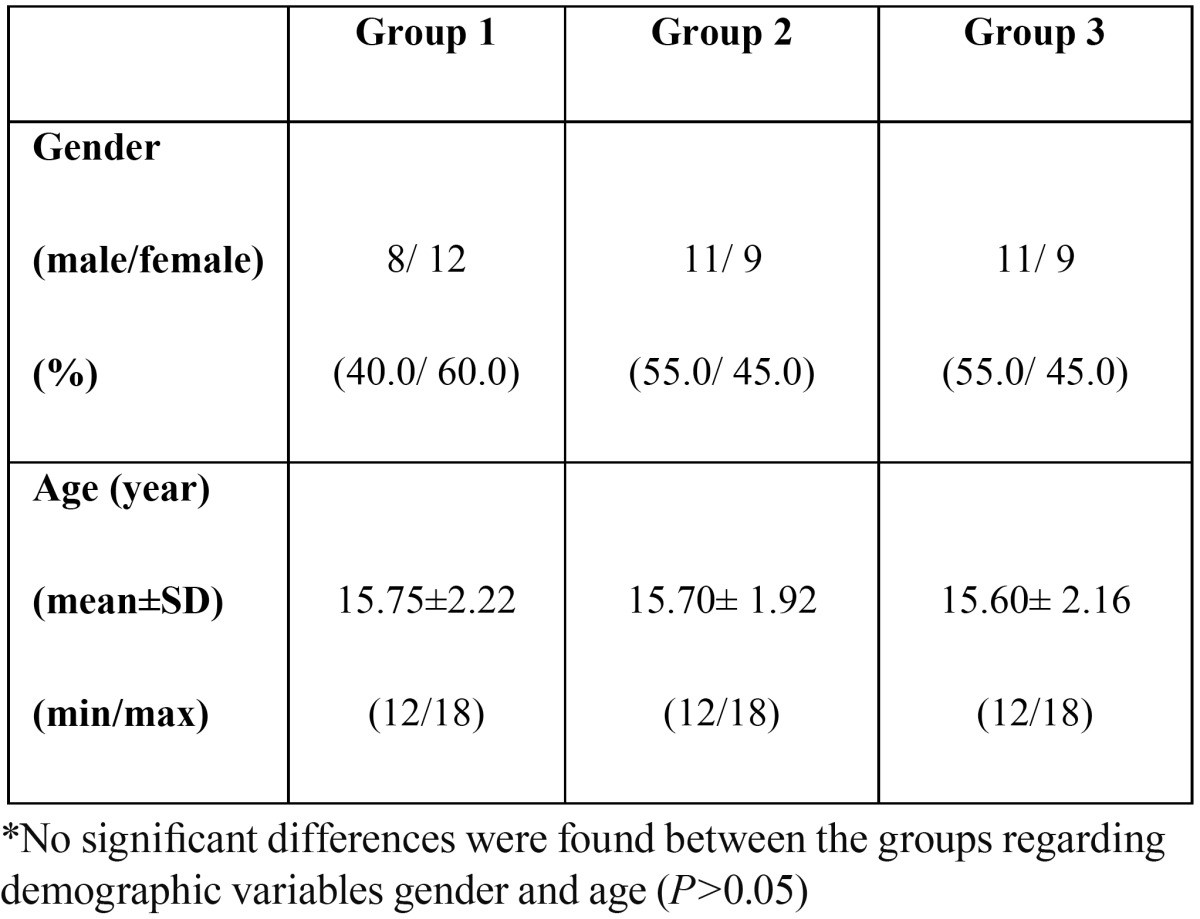
Demographic variables (n).

The inter and intra group comparisons are given in  Table 2.

**Table 2:**
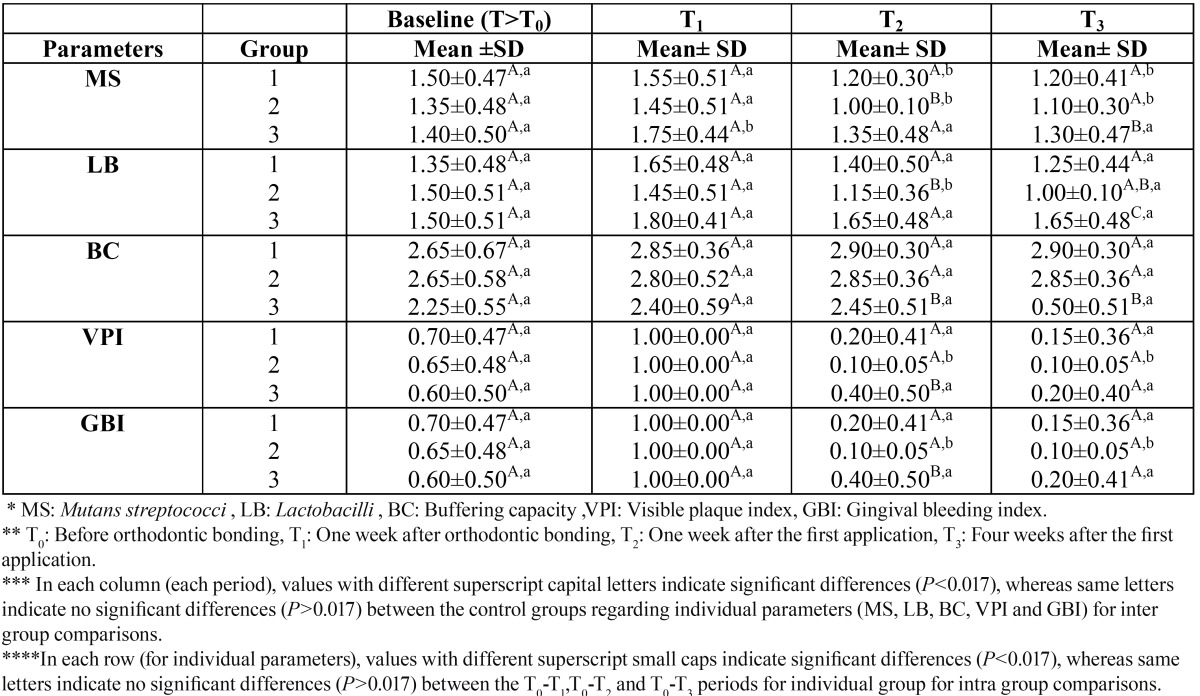
Inter and intra group comparisons for all groups.

-Intergroup Comparisons: No significantly different MS, LB, BC, VPI, and GBI levels were found at T0 and T1 among the groups (P>0.017).

More prominent decrease was seen in Group 2 regarding MS and LB levels than Group 1 (at T2) (P<0.017) and Group 3 (at T2 and T3, distinctly) (P<0.017). Group 1 resulted in lower MS and LB levels compared with Group 3 but these were not significantly different at T2 (P>0.017). Group 1 saw significantly decreased LB levels at T3 compared with Group 3 (P<0.017).

Groups 1 and 2 (at T2 and T3) (P<0.017) showed significantly higher BC values compared with Group 3.

Group 3 exhibited higher VPI scores compared with Groups 1 (T2; P<0.017, T3; P>0.017) and 2 (T2; P<0.017, T3; P>0.017).

Group 3 resulted in higher GBI scores compared with Groups 1 (T2; P<0.017, T3; P>0.017) and 2 (T2; P<0.017, T3; P>0.017).

-For Intragroup Comparison: In Group 1, MS levels significantly decreased at T2 and T3 compared with at T0 (P<0.017). Except for these comparisons, no significant differences were found between T0 and the other periods (T1, T2, and T3) with regard to MS, LB, BC, VPI, and GBI parameters (P>0.017).

In Group 2, MS levels significantly decreased at T2 and T3 compared with T0 (P<0.017). LB levels also significantly decreased at T2 compared with T0 (P<0.017). Additionally, significantly lower VPI and GBI scores were found at T2 and T3 compared with T0 (P<0.017). Except for these comparisons, no significant differences were found between T0 and the other periods (T1, T2, and T3) with regard to MS, LB, BC, VPI, and GBI parameters (P>0.017).

In Group 3, MS levels significantly increased at T1 compared with T0 (P<0.017). Except for these comparisons, no significant differences were found between T0 and the other periods (T1, T2, and T3) with regard to MS, LB, BC, VPI, and GBI parameters (P>0.017).

## Discussion

Fixed orthodontic appliances are known to have adverse affects on oral hygiene (8). Preventive measures such as good oral hygiene, noncariogenic dietary habits, and regular fluoride application should be carefully carried out throughout the treatment (7,8,13). Moreover, these attempts may not always be sufficient to avoid microbial colonization (7,8). Thus, adjunctive preventive applications i. e. applying chemotherapeutic agents, may be a key factor in reducing the potential risks of cariogenic microflora (7,14).

It is also important to provide better oral hygiene features by regular toothbrushing and flossing at home, particularly for the orthodontic patient (14). Therefore, in addition to professionally applied varnish treatments (1,4,5,7), brushing teeth regularly with Cervitec®Gel formulations (with respect to its usage indications) and monitoring in four-week recall periods could be considered as an average interval follow-up period for patients in our study. As previously stated (14), home-based self-application treatments may decrease the plaque accumulation around the bands and brackets. Because of the above reasons, the short-term (T2, one week; T3, four weeks) combined effects of Cervitec®Plus+Cervitec®Gel (Group 2) formulations were compared with one-visit varnish treatments in terms of MS, LB, BC, VPI, and GBI levels of orthodontic patients with fixed appliances.

This study demonstrated that combination therapy with Cervitec®Plus (one-visit application) and Cervitec®Gel (through toothpaste usage at home) is beneficial in reducing salivary bacterial counts and improving oral hygiene features compared with one-visit Cervitec®Plus and Fluor Protector® applications in up to four weeks.

Increased levels of MS and LB were commonly found in the oral cavity after bonding orthodontic attachments (14). In this study, the CRT chair-side test was used to measure MS, LB, and BC levels. Different studies have also supported this method (15,16). Additionally, a significant correlation had been found between conventional analysis with MSR agar and the MS or LB chair-site tests (17). To analyze the caries-associated parameters (MS, LB, and BC), a blind (had no idea about the treatment groups) experienced examiner conducted the test. This examiner collected all saliva samples and measured salivary bacterial counts and BC levels. VPI and GBI scores were presented as the key factors indicating the oral hygiene status of the patients (8,10,12). Thus, in order to screen the oral hygiene features of the patients, another blind examiner (initials of the blind examiner) measured these parameters.

According to the initial MS and LB measurements of this study, the study group could be considered a high caries risk group (>105) (15,16). However, T0 (baseline) and T1 values were not significantly different according to the MS, LB, BC, VPI, and GBI parameters among the groups (P>0.017). These findings could facilitate the data analysis among the groups, particularly at T2 and T3 periods. From this standpoint, undesirable individual responses to all treatments could be minimized. This is also concurrent with the randomly selected high caries risk group of the presented orthodontic clinic.

Increased MS levels in saliva and around the bonded teeth had been reported three months after bonding (8). In this study, both MS and LB levels increased compared with T0 values after the bonding period (T1), in all groups. This finding is consistent with the results of previous studies (7,8,14,16,17).

Fluoride treatment may be beneficial in eliminating the possible complications of fixed orthodontic treatment. However, a valid fluoride application regimen has not been described yet in terms of providing reasonable oral hygiene features in orthodontic patients (6). Previous reports indicated that fluoride and CHX combination therapy could be beneficial in increasing the bacterio-static effect of fluoride (5,7,8).

Furthermore, commercially available tooth gel formulations have been recommended to provide better oral hygiene features in patients undergoing orthodontic treatments with fixed appliances (1). CHX-based gel formulations have recently been found to decrease the gingival bleeding scores in patients with special care needs (18). However, the potential staining risk and temporary bitter taste of toothpaste formulations containing CHX must be carefully considered (19,20). In order to reduce the staining effect of CHX (1), reduce the concentration of CHX from 0.75% to 0.50% in dentifrice. They postulated that 0.50% CHX reduces the staining risk without compromising the its effectiveness against gingivitis and bleeding. However, Sari et al. (4) showed that although 0.2% CHX reduces S. Mutans levels, it does not have any effect on Lactobacillus count in saliva. Nevertheless, manufacturer instructions indicate that using Cervitec®Gel formulation for about four weeks can help avoid possible side effects, including staining of the teeth and temporary bitter taste problems. No staining or temporary bitter taste problems were recorded in this study. One-visit varnish treatment model was used to compare the potential use of CHX tooth gels over longer periods and to observe the exact desirable or undesirable effects of combined therapies for future studies.

The most common preventive application during fixed orthodontic treatments was repeated varnish treatments in different time intervals (8,14). Various varnish treatment procedures and frequencies have been documented in the literature regarding the use of Cervitec®Plus or fluoride varnishes in orthodontic patients (7,8,21). Moreover, fluoride varnish treatments were found to reduce salivary bacterial counts and decrease the plaque index and bleeding scores (22). Possible positive effects of applying repeated varnish treatments documented in the literature were reducing bacterial counts and plaque scores (2,7,8,21). However, findings which indicate that repeated applications did not always result in better antimicrobial suspension or acceptable oral hygiene features with evidence of long-term outcomes cause controversies (2,5). Previous findings revealed that MS suppression could persist after a single application of CHX-containing varnish for two weeks (21), three weeks (14), or six months (23). Additionally, such investigation designs or methodologies clearly showed that varnish treatment with CHX or therapeutic fluoride materials was used in various ways either before (intensive) or after the bonding procedures (7,8,21).

Moreover, an effective way to apply varnish immediately after the bonding procedure was found to be a main concern (21,24). Furthermore, in this study, because one-visit application procedures and combined therapy (to avoid the side effects of self application of CHX gel formulation) were utilized for short follow-up periods, an intensive application period was not used. However, if varnishes are used before bonding, similar to previous study designs (1,10), negative microflora alterations after the bonding procedure (T1) and the effect of combined therapy might be concealed since the varnishes could have a prolonged cumulative antimicrobial effect up to the T2 and T3 periods. In this way, the study aimed to evaluate the short-term effectiveness of combination therapy over one-visit varnish treatment models in a dental clinic, particularly after the bonding procedure.

Regarding intergroup comparisons, MS and LB levels in Group 2 decreased significantly better than Groups 1 (at T2) (P<0.017) and 3 (at T2 and T3, distinctly) (P<0.017). This evidence showed that the self-application of tooth gel formulation could increase the success of one-visit varnish treatments. This feature might be attributed to remaining CHX particles around negatively ionized tooth surfaces with regular tooth brushing (1,7,8,10,23). This study also confirmed that one-visit application of CHX-based varnishes (Group 1) showed better antimicrobial properties over the fluoride (Group 3) varnish treatments, as previously described (8,10,11). The limited/biased factor of Group 2 should also be taken into account, as it had a potential advantage since the home-based self-application of Cervitec®Gel could increase treatment success over professionally applied varnishes in terms of providing acceptable oral hygiene features. Furthermore, since the short-term present outcomes of varnish treatments (in Groups 1 and 3) were found to be less successful, sealing all areas with the varnishes should not be overlooked since a great deal of care was declared, as previously noted (25).

Nonetheless, the application of varnish treatments (in Groups 1 and 3) could be almost difficult when high viscosity features of these agents clearly obstruct their potential usage around bands and brackets after bonding procedures (24). The possible failure problems related to varnish treatments were documented as rapid recolonization and returning of MS bacteria to baseline levels (2,7,8,16,17,21) . In this study, Group 1 and, in particular, Group 2 exhibited significantly lower (P<0.017) MS levels in saliva at T2 and T3 compared with individual T0 values. Moreover, in Group 3, the lower but insignificant (P>0.017) MS levels were found at either T2 or T3 compared with individual T0 values. Considering LB bacteria, such differences were found compared with MS. Group 1 and, in particular, Group 2 exhibited lower but insignificant (P>0.017) LB levels compared with T0 values. Also, in Group 3 the LB levels were found almost higher at T2 and T3 compared with individual T0 values (P>0.017). After the varnish treatments, the chosen test periods (T2, one week and T3, four weeks) could also reflect risk of recolonization since this problem could be observed at about two weeks (21). These periods (one week and four weeks) may be considered worst-case scenario conditions for all groups. Therefore, after observing the above findings, the rapid bacterial recolonization feature is understandably more risky in Group 3 than in Groups 1 and 2, respectively ( Table 2). These findings may also be gained from the acceptable intragroup variations of the tested groups, which simplified screening of the effectiveness of therapeutic approaches and increased the power of the present study.

After application of the fixed orthodontic appliances, the destructive effect could be minimized only with the BC of the saliva. Moreover, the metallic bracket features have been shown to induce specific changes in the oral environment (26). In this study, the BC was found higher in Groups 1 and 2, and significantly different during T2 and T3 (P<0.017) compared with Group 3. Also, Group 2 exhibited higher BC values at T2 and T3 (P>0.017) compared with Group 1. These findings highly extrapolated that the use of a combined therapy would be beneficial, particularly during the early stages of the fixed orthodontic treatment. Recommending home-based CHX containing tooth gel formulations with professional varnish therapies seemed to improve oral hygiene features as shown by the BC results of this study.

Since increased plaque accumulation and gingival bleeding are known as possible side effects of fixed orthodontic treatments (27), this study also evaluated VPI and GBI values. Bonding teeth with metallic appliances increases plaque accumulation and gingival bleeding (28). In this study, the VPI and GBI scores tended to increase at T1 (immediately after bonding) compared with T0 for all groups (P>0.017), consistent with previous findings (29). Group 3 exhibited higher VPI and GBI scores compared with Groups 1 and 2 at T2 (P<0.017) and T3 (P>0.017). For Group 1, a positive effect was found, consistent with previous findings (10,29,30). Group 2 also experienced lower plaque accumulation and bleeding problems compared with Group 1 (P>0.017) at T2 and T3. In addition, in Group 2 the VPI and GBI scores were also found significantly lower at T2 and T3 compared with T0 (P<0.017). Considering all of the parameters, the possible beneficial effects of combined therapy over one-visit varnish applications (Groups 1 and 3) verified by the decreasing VPI and GBI results found in this study. Previous findings revealed that six or 12-weeks application of low concentrated CHX-based tooth gel formulations (0.50%) might be beneficial in reducing plaque accumulation and gingival bleeding without considering side effects such as staining and bitter taste during orthodontic treatment (1). In this study, reduced concentration of CHX (0.2%) tooth gel formulation with 0.2% sodium fluoride was used in a self-application procedure and was also found to be a promising agent for the prevention of decalcification in orthodontic patients. Findings of this study are in agreement with the previous CHX-based toothpaste studies (10). Although the one-visit varnish treatment procedure could increase the effectiveness in Group 2 compared with Group 1, the relatively beneficial prolonged effect of the present results at T3 of this study showed that Cervitec®Gel formulation could be a part of routine oral hygiene in orthodontic patients. From another perspective, further investigations are needed on a more frequent, home-based self-application procedure combined with repeated varnish treatments over a long evaluation period.

The measured parameters being better in Group 2 than Groups 1 and 3 may also be explained by the Hawthorne effect. Children were highly motivated when they were told to brush their teeth with the specific gel formulation in Group 2. Thus, recommending home-based preventive applications instead of routine toothbrushing practices in different time intervals could be useful in motivating children undergoing fixed orthodontic treatment. This may have been a confounding factor, as previously described (10).

Overall, within the limitations of this study, the combination of chlorhexidine varnish and chlorhexidine-fluoride containing dentifirice was found to be a successful and promising alternative for improving oral hygiene features of patients lacking sufficient oral hygiene characteristics during orthodontic treatment. Moreover, the possible side effects of toothpastes must be carefully monitored and, if required, eliminated by the clinician (1,10).

One-visit chlorhexidine varnish treatment combined with a home-based chlorhexidine-fluoride gel formulation may be implemented as a caries prevention strategy, for patients undergoing fixed appliance treatment and particularly under high risk of caries.

Further studies are needed to evaluate long-term effectiveness of repeated preventive procedures.
